# National immunization technical advisory groups (NITAGs) in the WHO Eastern Mediterranean Region (EMR): A decade of shaping immunization policies, 2010–2021

**DOI:** 10.1016/j.vaccine.2023.12.049

**Published:** 2024-01-25

**Authors:** Gerald Etapelong Sume, Hasan Quamrul, Sharifuzzaman Md, John Kissa, Yvan Hutin

**Affiliations:** a Immunization Vaccine Preventable Disease and Polio Transition Unit, Department of Communicable Diseases, WHO Regional Office of the Eastern Mediterranean Region, Cairo, Egypt; b Department of Communicable Diseases, WHO Regional Office of the Eastern Mediterranean Region, Cairo, Egypt

**Keywords:** National Immunization Technical Advisory Groups (NITAGs), Immunization policy decisions, Eastern Mediterranean Region, NITAG functionality

## Abstract

In the Eastern Mediterranean Region (EMR) of the World Health Organization (WHO), little is known on National Immunization Technical Advisory Groups’ (NITAGs) outputs, including recommendations and their outcomes. We abstracted information from the WHO/UNICEF joint reporting forms and extracted implemented immunization policy decisions from the WHO immunization portal. We describe trends in establishments and functionality of NITAGs and immunization policies implemented in EMR from 2010 to 2021. In 2013, all 22 EMR countries had a NITAG, although only 20 remained active in 2021. The number of countries meeting six NITAG process indicators increased from 7 in 2010, to 14 in 2019, then reduced to 12 in 2021. In 2021, the proportion of countries with a functional NITAG decreased with income level, from 83% in high-income countries, to 55% in middle-income countries and 20% in low-income countries. From 2010 to 2021, there were 103 new vaccine introductions, 31 vaccine switches, and 28 schedule changes implemented across all 22 countries, irrespective of income groups. While NITAGs are established and making recommendations in countries, their functionality decreases with income level. Governments should continue to invest in NITAGs, including on strengthening processes and ensuring that recommendations made are based on evidence to decision frameworks.

## 1 Introduction

NITAGs are multidisciplinary groups of national experts who provide technical advice to the Ministry of Health (MoH) on immunization policies [1]. They make recommendations to governments based on evidence. Governments consider political, financial, and social context in prioritizing implementation of those recommendations. They are to national governments what the Strategic Advisory Group of Experts on Immunization (SAGE) and Regional Immunization Technical Advisory Groups (RITAGs) are to the global and regional levels, respectively.

In the 1970s, countries formally started to institutionalize national immunization programmes to provide lifesaving vaccines to their children and reduce the incidence of vaccine preventable diseases. In the 1980s, some countries had official bodies that would advise the MoH on immunization policy decisions. However, those where not structured and usually not independent. Immunization policy making includes introduction of new vaccines or updating existing policies (for example, switching from a vaccine already in use to another for the same disease or changing the schedule for a vaccine already in the national immunization programme). In 2010, WHO formalized the structure and reporting of National Immunization Technical Advisory Groups (NITAGs) through the WHO/UNICEF joint reporting form (JRF). In a 2017 World Health Organization (WHO) position paper, the SAGE stressed the importance of NITAGs as a core institution of well-functioning immunization programmes [2]. It also urged countries, WHO, partners and donor communities to continue providing support and facilitate the work of NITAGs. The 2020 Global Vaccine Action Plan (GVAP) goal stated that all countries should create and strengthen national capacity to formulate evidence-based policies [1], [2]. The monitoring and evaluation framework of the immunization agenda 2030 (IA2030) identifies NITAGs as one of the independent technical review bodies that can enforce country ownership and accountability [3].

In 2019, globally, 158 countries reported that they had a NITAG [4]. Overall, the number of NITAGs that meet the six NITAG functionality criteria (NITAG process indicators) increased from 41 in 2010, to 120 in 2019 [5]. These process indicators are: (i) having a formal written term of reference; (ii) having a legislative or administrative basis for the creation of the NITAG; (iii) having at least five main expertise areas represented in core membership; (iv) at least one meeting held each year; (v) circulating meeting agenda and background documents at least one week before meetings; and (vi) use of a mandatory disclosure of interests by members. Multi-partner initiatives across different regions of WHO made this possible [6], [7], [8], [9]. The Global NITAG Network (GNN) created by the Supporting Immunization and Vaccine Advisory Committees (SIVAC) Initiative is one of such initiatives that consists of a platform to share NITAG experiences and best practices [10]. The GNN promotes networking and capacity building through workshops and use of the NITAG resource centre [11].

The WHO Eastern Mediterranean Region Vaccine Action Plan (EMRAP) 2016 to 2020 provided a framework for the implementation of GVAP, taking into consideration regional priorities and contexts [12]. The region was committed to establishing and strengthening independent NITAGs to help formulate evidence-based immunization policies. In 2010, 19 (86 %) countries in the Eastern Mediterranean Region (EMR) reported the existence of a NITAG. Of those, 7 (37 %) met the six NITAG functionality criteria [13]. In 2012, of all WHO regions, EMR had the highest proportion of countries with NITAGs with legislative basis and NITAGs that met all functionality criteria [14]. From 2010 to 2012, EMR countries with NITAGs increased by 11 % and countries with functional NITAGs increased by 86 %. However, no review subsequently examined these trends in EMR. While several publications documented NITAG establishment and functionality in the EMR, little is known on the output and outcome of immunization policy recommendations made by NITAGs to MoH. In this paper we describe trends in establishment and functionality of NITAGs, and immunization policy decisions implemented in countries of the Eastern Mediterranean Region of the World Health Organization from 2010 to 2021.

## 2 Methods

***Regional context***

The EMR is made of 21 member states (Afghanistan, Bahrain, Djibouti, Egypt, Iran, Iraq, Jordan, Kuwait, Lebanon, Libya, Morocco, Oman, Pakistan, Qatar, Saudi Arabia, Somalia, Sudan, Syria, Tunisia, United Arab Emirates and Yemen) and the Occupied Palestinian Territory (including East Jerusalem), henceforth referred to as Palestine. According to the United Nations Population Division, the total population in WHO EMR in 2021 was 744,601,716 inhabitants [15]. This review analyzed data transmitted by these 21 Member States and Palestine.

***Study design and period***

We conducted a retrospective review for the period of 2010 to 2021.

***Data collection***

**Joint reporting form.** We extracted information from the WHO immunization data portal [16] on information elements related to the work of NITAGs. Member States had reported this information alongside other annual (January to December) immunization data using the JRF [17] from 2010 to 2021. A designated focal person from the MoH enters the data and submit to WHO through the regional offices. The submitted data is reviewed by WHO and feedback on identified errors provided to countries. It is for the country to update the information or not. The NITAG information for some countries was not available in the WHO immunization portal. Some of these countries that did not submit the JRF within the stated window of a given year, went ahead to share partially or completely filled JRF spreadsheet with the regional office after the deadline. We used such JRF spreadsheets available in the archives of the Eastern Mediterranean Regional Office (EMRO) of WHO and WHO headquarters (WHO HQ) to complete missing reports/NITAG information in the data extracted from the WHO immunization portal. The reporting of NITAG indicators in JRF started in 2010 with some indicator updates over time. The most recent update in JRF took place in 2021.

**WHO immunization data portal.** We also extracted data on implemented immunization policy decisions from the WHO immunization data portal. These included new vaccine introductions, vaccine switches, and change in national immunization schedule. We shared the data extracted from the WHO immunization portal on implemented immunization policy decisions with all EMR countries for review, update, validation, and feedback.

***Income groups***

We used the World Bank classification of income level to categorize countries by income group from 2010 to 2021, pulling together the lower and upper middle income countries categories into middle income countries (MIC). From 2010 to 2016, there were six (Bahrain, Kuwait, Oman, Qatar, Saudi Arabia, United Arab Emirates) high income countries (HIC), 14 MIC and two (Afghanistan and Somalia) low-income countries (LIC). While the HIC category remained stable, Yemen and Syria dropped from MIC to LIC from 2017 to 2021, and Sudan from MIC to LIC from 2019 to 2021 [18]. We used Gavi archives at EMRO to classify countries according to their eligibility to Gavi over the same period. From 2010 to 2020, there were 6 Gavi eligible countries (Afghanistan, Djibouti, Pakistan, Somalia, Sudan, and Yemen) and 16 Gavi non-eligible countries in EMR. Syria became Gavi eligible in 2021, making 7 countries.

***Definition and criteria***

Functionality of NITAGs

We considered NITAGs as functional when they met the six NITAG process indicators as per WHO guidelines and used in other publications [3], [15], [19], [20]. These included (i) formal written term of reference; (ii) a legislative or administrative basis; (iii) at least five main expertise areas represented in core membership; (iv) at least one meeting held each year; (v) circulation of the agenda and background documents at least one week before meetings; and (vi) use of a mandatory disclosure of interests by members.

Implemented immunization policy decision

We considered an implemented immunization policy decision as an effective change in the immunization programme between 2010 and 2021 that constituted in any of the following: (i) new vaccine introduction (NVI): introduction of a new vaccine in the immunization programme; (ii) vaccine switch: when a vaccine already in use in the immunization programme was changed to another to protect against the same disease; and (iii) immunization schedule change: when there is a change (addition and/or subtraction) in the number of doses or change in age of administration for a vaccine already introduced in the programme.

### 2.1 Data analysis

We created a database to regroup the extracted data. A NITAG was considered functional if it met all six functionality criteria for a given year and over the review period. We analyzed trends in NITAG process indicators, NITAG functionality and implemented immunization policy decisions. We compared NITAGs across income group and Gavi eligibility status in terms of functionality and number of implemented immunization policy decisions (new vaccines introduced, vaccine switches and national immunization schedule change). In each income group and Gavi eligibility status group, we divided the total number of immunization policy decisions implemented from 2010 to 2021 by the average number of countries to calculate the ratio of implemented immunization policy decisions per country in each category. We compared the ratio of implemented immunization policy decisions across income group and Gavi eligibility status categories.

## 3 Results

Of the expected 264 JRF country reports from 2010 to 2021, we obtained 257 (97 %); 229 directly from WHO immunization portal and 28 JRF spreadsheets from EMRO and WHO HQ archives. Of the 257 reports found, 13 had no information reported on NITAGs. The overall NITAG information completeness in the JRF was 244 (92 %).

***Presence of NITAGs***

**Fig. 1:**
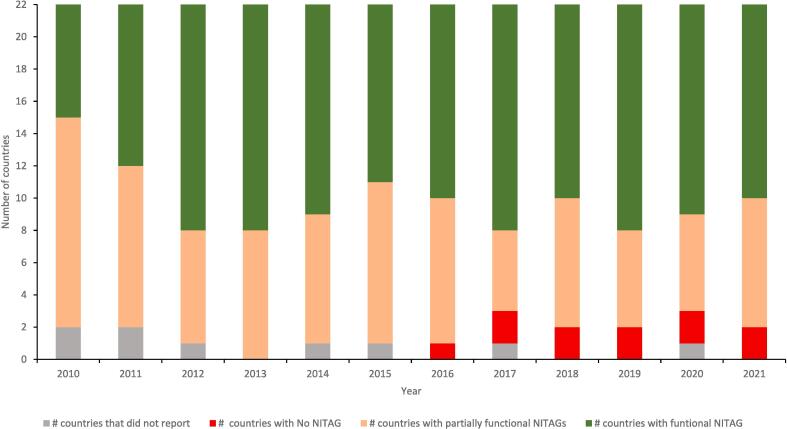
Number of countries with functional National Immunization Technical Advisory Groups (NITAGs), EMR, 2010–2021.

Twenty countries reported having a NITAG in 2010 and 2011. This increased to 21 in 2012, peaked at 22 countries in 2013, and decreased to a minimum of 19 (86 %) in 2017 and 2020. Overall, between 2010 and 2021, the average number of countries with NITAGs was 20 (Fig. 1). Somalia and Lebanon last reported on their NITAGs in 2015 and 2016 respectively.

***Functionality of NITAGs***

Between 2010 and 2021, the average number of countries with functional NITAGs was 12. There was progressive increase in the number of countries with a functional NITAG from 7 in 2010, a peak at 14 in 2012 and 2013 and a decrease to 13 in 2014 (Fig. 1).

**Fig. 2:**
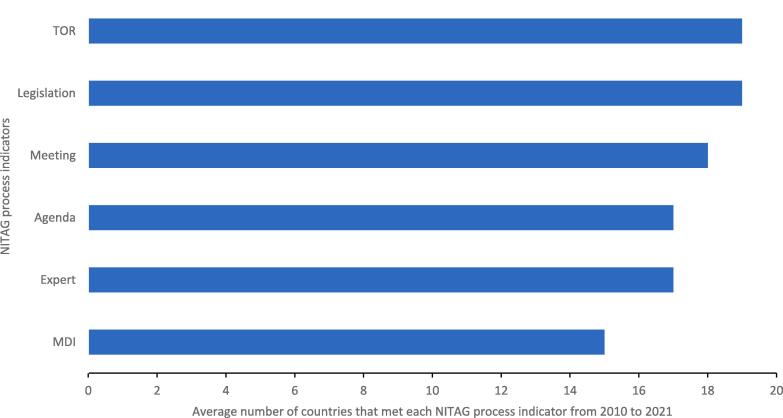
Average number of countries that met key NITAG process indicators, EMR, 2010–2021(TOR: has a formal written term of reference; Legislation: has a legislative or administrative basis creating the NITAG; Meeting: holds at least one meeting each year; Agenda: circulates agenda and background documents at least one week before meetings; Expert: has at least five main expertise areas represented in core membership and, MDI: implements mandatory disclosure of interests by members.).

From 2010 to 2021, on average, 19 countries had formal written terms of reference and administrative basis for the creation of their NITAG and 15 implemented mandatory disclosure of interests by members (Fig. 2). From 2010 to 2021, there was an increase in the proportion of countries with at least five areas of expertise represented in core membership (55 % to 82 %), NITAGs with a legislative basis (77 % to 91 %) and NITAGs that held at least one meeting (73 % to 77 %). The proportion of countries for which NITAGs had formal terms of reference and with mandatory disclosure of interest remained constant at 91 % and 59 % respectively. However, over the same period, there was a drop in the proportion of countries whose NITAGs circulated agenda and background documents at least one week before meetings (77 % to 73 %). Five countries who had earlier reported having a term of reference or legislative basis failed to do so at least once in subsequent years.

From 2010 to 2021, the proportion of countries with functional NITAGs was highest in HIC for nine out of the 12 years reviewed and lowest in LIC for 11 out of the 12 years reviewed. In 2021, the proportion of countries with a functional NITAG decreased with decreasing income group, from five (83 %) of six HICs, to six (55 %) of 11 MICs, and one (20 %) of five LICs. In 2021, nine (60 %) of 15 NITAGs in countries not eligible to Gavi were functional compared with three NITAGs (43 %) out of seven Gavi eligible countries.

***Implemented immunization policy decisions***

**Fig. 3:**
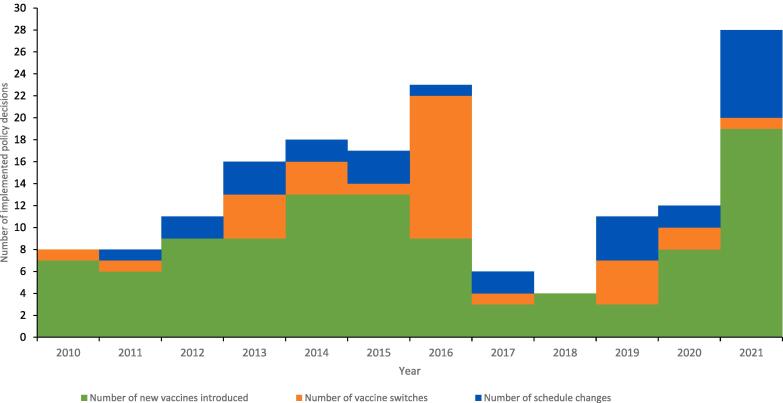
Number of implemented immunization policy decisions, EMR, 2010–2021 [These are immunization policy decisions effectively implemented by countries (not recommendations) from 2010 to 2021].

There was a total of 162 implemented immunization policy decisions from 2010 to 2021 in EMR. Of these, 103 (64 %) were new vaccine introductions, 31 (19 %) were vaccine switches and 28 (17 %) were national immunization schedule changes. The number of implemented immunization policy decisions averaged at 14 per year. New vaccine introductions peaked at 19 in 2021 in 17 countries, vaccine switches peaked at 13 in 13 countries in 2016 and change in immunization schedule peaked at eight in 2021 in seven countries (Fig. 3). Overall, the highest number of implemented immunization policy decisions was 28 in 2021 across 19 countries. Five countries introduced COVID-19 vaccine in 2020 with another 17 (77 %) in 2021. The most frequent vaccine switch was trivalent oral polio vaccine (tOPV) to bivalent oral polio vaccine (bOPV) (n = 13, 42 %). The most frequent schedule change was injectable polio vaccine (IPV) related (n = 14, 50 %).

By 2021, all HIC (n = 6, 100 %) had introduced hepatitis B birth dose and pneumococcal conjugate vaccine (PCV), five (83 %) had introduced rotavirus vaccine and one (17 %) human papilloma virus vaccine (HPV). Likewise, by 2021, 73 % (eight on 11) MIC had introduced hepatitis B birth dose and PCV, seven (64 %) had introduced rotavirus vaccine and one (9 %) HPV. Except for Syria that had introduced hepatitis B birth dose vaccine in 2003 when it was a MIC, no LIC introduced any of the priority vaccines before 2010. By the end of 2021, 60 % (three on five) LIC had introduced PCV and rotavirus vaccine, two (40 %) had introduced hepatitis B birth dose and none for HPV. No Gavi country introduced PCV, HPV and rotavirus vaccine before 2010 (Table 1).

**Table 1:** Number of countries that introduced high priority vaccines by income group and Gavi status, EMR, 2010–2021.^1^

	**By income group**^2^			**By Gavi status**		
**Vaccines**	**LIC**	**MICs**	**HICs**	**Gavi**	**Non-Gavi**	**Total**
	**#/Total (%)**	**#/Total (%)**	**#/Total (%)**	**#/Total (%)**	**#/Total (%)**	**#/Total (%)**
**Hepatitis B birth dose**	1/4 (25 %)	2/5 (40 %)	3/3(100 %)	2/6(33 %)	4/6(67 %)	6/12(50 %)
**HPV**	0/5 (0 %)	1/11 (9 %)	1/6(17 %)	0/7(0 %)	2/15(13 %)	2/22(09 %)
**PCV**	3/5 (60 %)	8/11 (73 %)	1/1(100 %)	5/7(71 %)	7/10(70 %)	12/17(71 %)
**Rotavirus**	3/5 (60 %)	7/11 (64 %)	3/4(75 %)	5/7(71 %)	8/13(62 %)	13/20(65 %)

#: Number; PCV: Pneumococcal conjugate vaccine; HPV: Human papillomavirus vaccine.

1 These are immunization policy decisions effectively implemented by countries (not recommendations) from 2010 to 2021. In each category, only countries that had not introduced the vaccines of interest by 2010 are considered in the denominator. Before 2010, 5HIC, 3HIC and 2HIC had introduced PCV, Hepatitis B birth dose and Rotavirus vaccine respectively. Before 2010, 6 MIC introduced Hepatitis B birth dose and none introduced PCV, Rota or HPV. As at end 2021 in EMR, there were 6 HIC, 11 MIC, and 5 LIC. There were also 15 countries not eligible to Gavi and 7 Gavi eligible countries. HIC: High Income Countries; MIC: Middle Income Countries; LIC: Low Income Countries.#: Number; PCV: Pneumococcal conjugate vaccine; HPV: Human papillomavirus vaccine.

2 HIC: High Income Countries; MIC: Middle Income Countries; LIC: Low Income Countries.

From 2010 to 2021, a median of five new vaccines were introduced, a median of one vaccine were switched and a median of one schedule change occurred. Sudan (LIC) and Pakistan (MIC) are the only Gavi eligible countries with new vaccine introductions more than the median. Sudan and Somalia, both LIC and Gavi eligible implemented more than one schedule change. Four MIC (Iraq, Libya, Morocco, and Pakistan) and four HIC (Bahrain, Kuwait, Oman, and United Arab Emirates) reported more than the median vaccine switches over this period (Table 2).

**Table 2:** Total new vaccines introduced, vaccine switches and schedule change by income group and Gavi status, EMR, 2010 – 2021.^1^

**Outcome of interest**	**By income group**			**By Gavi status**		
	**LIC**	**MICs**	**HICs**	**Gavi**	**Non Gavi**	**Total**
	**#/*Total* (%)**	**#/Total (%)**	**#/Total (%)**	**#/Total (%)**	**#/Total (%)**	**#/Total (%)**
**Number of countries with new vaccine introductions (NVI) > median**	1/5(20 %)	3/11(27 %)	1/6(17 %)	2/7(29 %)	3/15(20 %)	5/22(23 %)
**Number of countries with schedule changes (SC) > median**^2^	2/5(40 %)	2/11(18 %)	3/6(50 %)	2/7(14 %)	5/15(33 %)	7/22(32 %)
**Number of countries with vaccine switches (VS) > median**	0/5(0 %)	4/11(36 %)	4/6(67 %)	1/7(14 %)	7/15(47 %)	8/22(36 %)

1 As at end 2021 in EMR, there were 6 HIC, 11 MIC, and 5 LIC. There were also 15 countries not eligible to Gavi and 7 Gavi eligible countries.

2 Median for new vaccines introductions (NVI) = 5; Median for vaccine switches (VS) and schedule change (SC) or schedule change = 1.

There was little variation in the ratio of implemented immunization policy decisions across income groups and Gavi eligibility status over the review period. From 2010 to 2021, a total of 52 immunization policy decisions were implemented by 6 HIC, (ratio 9:1). This consisted in five new vaccine introductions, two vaccine switches and two schedule changes. Over the same period, 90 immunization policy decisions were implemented by an average of 13 MIC (ratio 7:1). These were 5 new vaccine introductions, 1 vaccine switch and 1 schedule change. From 2010 to 2021, 20 immunization policy decisions were implemented by an average of 3 LIC, (ratio 7:1). These were 4 new vaccine introductions, 1 vaccine switch and 2 schedule changes. From 2010 to 2021, 49 and 113 immunization policy decisions were implemented by Gavi eligible (ratio 8:1) and Gavi non-eligible (ratio 7:1) countries respectively.

## 4 Discussion

From 2010 to 2021, all EMR countries reported the existence of a NITAG at least once, but the functionality varied over time, by income group and by Gavi status. Over a hundred new vaccine introductions occurred, with several switches and national immunization schedule changes. These did not vary across income groups and Gavi status although HIC were faster with the introduction of priority vaccines.

In 2013, four years after countries started reporting on NITAG indicators in the JRF, all 22 EMR countries reported a NITAG, with 14 meeting all functionality criteria, and an improvement in the process indicators between 2010 and 2021. Efforts made by WHO and partners to support NITAG creation and operationalization are likely to explain this improvement. In 2008, EMRO conducted a situational analysis and prepared a guide for NITAG creation, functioning and developed a work plan to strengthen NITAGs [21]. Global and regional partnership also played a role in establishing and strengthening NITAGs in the region [9], in line with multi-partner initiatives in other WHO regions [6], [7], [8], [20]. From 2017 to 2021, NITAGs from Lebanon and Somalia, that once existed struggled to remain functional. Humanitarian, political, and socio-economic crisis faced by these countries may explain this disruption. Similar situations have been reported in the past where NITAGs seized to exist or function due to political turmoil or in times of crisis. However, when NITAGs had strong legislative basis and process, they can resume their functions after disruptions [6].

The functionality of NITAGs improved with higher income groups. NITAG functionality was also lowest in Gavi eligible countries, most of which were LIC. HIC are more stable, have stronger immunization programmes, stronger health systems and have the necessary financial and skilled human resources for their NITAG secretariat. A global review also reported a higher proportion of functional NITAGs in HICs [13], [14]. Several factors have been described to negatively affect the establishment and functioning of NITAGs and their secretariat. These include lack of funding, lack of work plans and agendas, lack of policy to manage interest, lack of human resources, insufficient training on evidence-based review processes, language barriers, limited access to critical literature and publications, and limited recognition of the NITAG by MoH [2], [5], [10], [22]. In addition, in the EMR, insecurity and humanitarian crises in about 50 % of countries challenged the GVAP goals and may have also affected the ability of NITAGs to play their role in some countries [23]. Nonetheless, despite the lower functionality, it’s been shown that NITAGs in low- and middle-income countries remain valuable in promoting evidence-informed decision-making, with the capacity to do more if the above-mentioned challenges are addressed [24].

Even though NITAG functionality varied by income and Gavi eligibility status, this did not seem to affect the number of implemented immunization policy decisions over the 12 years review period. HIC countries introduced priority vaccines faster. However, other countries, especially the LIC caught up with implemented immunization policy decisions whether in terms of new vaccines introduced, vaccine switches or immunization schedule changes over time. Some implemented new vaccine introduction in LICs may have been influenced by the availability of Gavi support to cover the cost, irrespective of the technical dossiers for decision making. However, the absence of a well mounted evidence-based case could affect sustainability in the long term when Gavi support is discontinued. New vaccine introductions in LIC Gavi eligible countries may also have been influenced by global and regional directives. Examples include the large number of vaccine switches in 2016 (global recommendation to switch from trivalent oral polio vaccine to bivalent oral polio vaccine) and the peak of new vaccines introduced in 2021 (COVID-19 vaccine as a response to the pandemic). HIC were the first to introduce COVID-19 vaccine (5 in 2020), while the others introduced in 2021. With multi-partner support, countries implemented these global emergency guidelines with immediate effect, irrespective of their income group and Gavi eligibility status. Following such emergency global guidelines, NITAGs usually just endorse the global recommendations without conducting their specific country evidence-to-recommendations processes. This unprecedented wave of vaccine introduction (within a year) as regards COVID-19 vaccine contrasts with the speed of introduction of other priority vaccines (over a decade), including hepatitis B birth dose, PCV, and rotavirus vaccine. Several factors can explain the delays in vaccine introductions. These include financial constraints [25] or delays in making policy decisions [26].

The present paper has two main limitations. First, the use of WHO/UNICEF JRF data exposed us to non-submission or data quality gaps on submitted report especially reporting inconsistencies as regards legislative basis for NITAGs and existence of terms of reference. These may lead to over estimation or under-estimation of the parameters analyzed. We were able to increase data completeness in our analysis from 87 % obtained from the WHO immunization portal to 97 % after review of JRF archives at regional and global levels. This limitation is unlikely to affect our interpretation of the trends and patterns. Second, we did not have a direct source of information on the actual recommendations formulated by the NITAGs. This prevents us from documenting the actual output of the NITAGs. To address this issue, we used implemented immunization policy decisions as a proxy indicator. Also, from 2021, WHO added two new indicators in the JRF, that is, information on recommendations made by NITAGs and recommendations adopted by the MoH. In 2022, 13 EMR countries reported that their NITAGs made at least one recommendation in 2021 and 12 reported that the MoH implemented at least one NITAG recommendation. Being a yes/no question, this added information does not describe the type and number of recommendations made and immunization policy decision implemented per country.

This review led to three main conclusions. First, in 2010–2021, investment led to a progression of the NITAG agenda in EMR. Second, NITAGs vary in their capacity and essential requirements. Third, the contribution of the NITAGs to the implementation of evidence-based policy requires further documentation. Based on these conclusions, we identified a few ways forward. First, governments should continue investing in NITAGs and WHO Regional office should build capacity on NITAG operations and proper reporting of NITAG variables in the JRF. Second, the NITAG secretariat should improve quality and decision-making process on immunization. This should be particularly important in LICs and MICs to ensure the sustainability of vaccine introductions after discontinuation of Gavi support. Third, NITAG secretariats should properly document the formulation of recommendations, along with the process used and the subsequent policy uptake. Implementation of these recommendations will ensure that we can build on what has been achieved with strengthening of NITAGs so that we can take the function of these precious national bodies to the next level.

## Declaration of competing interest

The authors declare that they have no known competing financial interests or personal relationships that could have appeared to influence the work reported in this paper.

## Data Availability

Data will be made available on request.
